# Development of student intent-based educational chatbot system with adaptive and attentive DTCN on symmetric convolution approach

**DOI:** 10.1016/j.mex.2025.103542

**Published:** 2025-08-05

**Authors:** Atul Kathole, Suvarna Patil, Dr. Devyani Jadhav, Hirkani Pathak, Amita Sanjiv Mirge

**Affiliations:** a Department of Computer Engineering, Dr. D. Y. Patil Institute of Technology, Dr. D. Y. Patil Dnyan Prasad University, Pimpri, Pune 411018, India; b Department of AIML, Set Sanjivani University, Kopargaon, Mumbai, 423601, India; c Assitant professor, Department of Computer Science and Engineering, Shri Ramdeobaba College of Engineering and Management, Ramdeo Tekdi, Gittikhadan, Katol Road, Nagpur 440013 Maharashtra, India; d Department of Computer Engineering, D.Y. Patil College of Engineering, Akurdi, Pune, 411018, India

**Keywords:** Chatbots, Intent classification, Bidirectional encoder representations from transformers, Transformer network, Text convolutional neural network, Averaging-based driving training-barnacles mating optimizer, Adaptive and attentive deep temporal convolutional network with symmetric convolution

## Abstract

The Accessing academic information like exam time tables, exam scores, syllabus and tutor information on institutional websites can be time consuming and tedious on behalf of the student. In this case, we will solve this dilemma by creating an intelligent and an automated Student Intent-based Educational Chatbot that integrates deep learning along with state-of-art optimization algorithms. Following the pre-processing of student inquiries with generic chatbot data sets, the system starts operating. The text data that were cleaned are represented as vectors through three high-performing language representations, i.e., BERT, TransformerNet, and Text CNN. Such vectors are subject to weighted selection of features and fusion where best features are selected and fused together by Averaging-based Driving Training -Barnacles Mating Optimizer (ADT -BMO) is a new hybrid metaheuristic optimization algorithm. ADT-BMO is smart when it comes to weighting the feature and optimization parameter to maximize the relevance of fused features. Thicken with Symmetric Convolution (AA-DTCN-SC) and the down-stream adaptive feature set refined above are fed to this network to achieve accurate intent recognition. ADT-BMO also improves AA-DTCN-SC model by optimizing the parameters hidden neurons, activation functions and epochs under which the classification accuracy is high. In testing the noted purpose, there is an automatic building of system responses to queries formulated using contextually right answers. The experimental simulations also illustrate the effectiveness of the superiority of this approach as the chatbot performs better than its baseline models of DTCN, RNN and Bi-LSTM by 4.44%, 3.3%, 10.59%, 11.9, respectively. The given research therefore presents a well-functioning, scalable and time-saving educational chatbot, which improves student engagement through provision of quick, precise, and pertinent scholarly assistance.

• To help save the time and work required of students and administrators by replying to common academic questions through the creation of an automated intelligent chatbot that is able to find correct and instant answers based on deep learning and NLP approaches.
• In order to eliminate the shortcomings of current chatbot platforms, i.e. irrelevant outputs and fabricated predictivity of user intent, through implementation of a different novel model (AA-DTCN-SC) on deep learning, which is optimized using a hybrid ADT-BMO algorithm to aid intent inference and user interaction in educational settings.

## Graphical abstract

**Figure:**
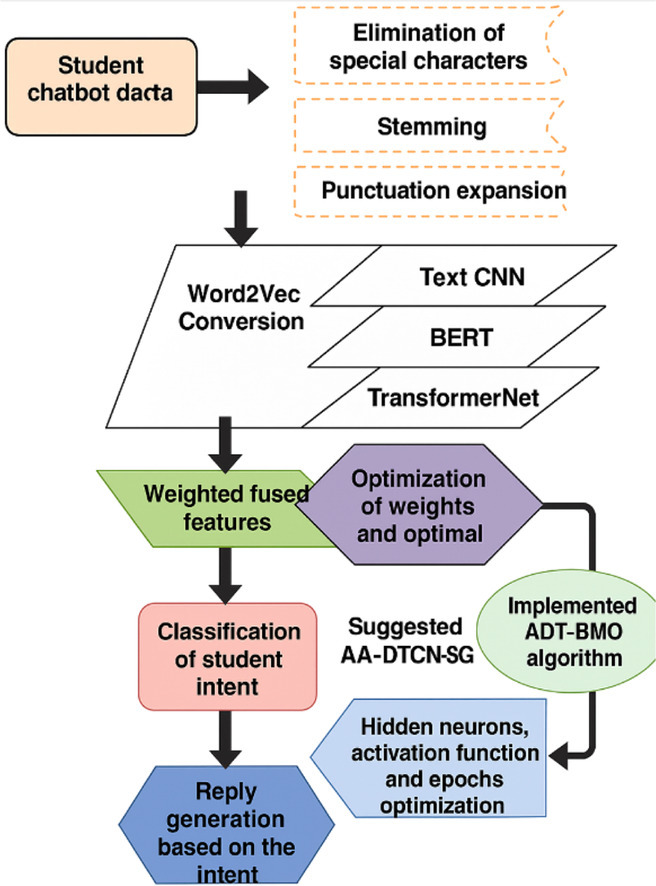

**Specifications table**

**Table:** 

**Subject area**	Computer Science
**More specific subject area**	Machine Learning
**Name of your method**	Adaptive and Attentive Deep Temporal Convolutional Network with Symmetric Convolution (AA-DTCN-SC)
**Name and reference of original method**	None
**Resource availability**	None

## Background

The development of both software and hardware and computers found its vast application in fields like locomotives, communication, healthcare, business, educational sectors, and many other industries [9]. In chatbots, the utilization of software computers helps both the customers and the manufacturers. At present, the chatbot is the most widely used method of chatting in online sources, as, even with normal human-used language, also known as Natural Language (NL), it allows for user-friendly interactions between humans and machines [4]. But, the difficulties undergone in the development of the chatbot are well concealed as it remains unknown to most people [10]. Chatbots strive to mimic human interactions as normally as it is by perfectly hiding their complex process to mimic so. An automatic method that aids the users in providing the necessary reply to their queries is called a chatbot [11]. They give organizations a better method to interact with their clients and boost their satisfaction of the client. They provide clients with an improved and more practical approach to obtaining their queries answered without the need to wait on the phone or without sending numerous emails to get the required response from the organizations [12]. Semantic web and Natural Language Understanding (NLU) approaches are used by chatbots to collect pertinent and helpful data from Knowledge Bases (KB) in terms of connected data. Various chatbots are available at present times [5]; however, their development necessitates a huge expense, and it is difficult to produce a huge volume of training data. The development of chatbots and the growth of new research regarding chatbots are rising as the connected data rise nowadays [13]. They continue to struggle with a variety of issues, like the classification of user intentions, language issues, numerous KBs, and the comprehension of analytical queries [6].

The old tutor-centered method still predominates in developed nations, where the students struggle to maintain pace with, retain knowledge, and manage the fast-paced nature of contemporary educational methods [14]. But, to fully utilize the potential of Information and Communication Technologies (ICT), the above-mentioned strategies fail in meeting the demands of the current digital age [15]. In order to enhance and fine-tune the learning experiences of their students, tutors should employ contemporary ICT techniques. Considering this, the application of popular and advanced technology has led to an important solution in solving this situation [16]. AI chatbots, which utilize conversational AI technology, serve as the foundation of a significant new digital educational system. With the help of the NL, AI chatbots are capable of communicating with humans in a variety of ways; thus, it founds their application in the many daily activities of humans [17]. They are typically employed by huge organizations for providing customer service for businesses or as virtual personal assistants. AI chatbots in an educational environment can serve as digital instructors, which can deliver study materials, encourage interaction with students and conversation provides suitable student feedback, and much more [18]. By providing replies to students' queries and offering instruction around-the-clock, AI chatbots can serve as a supportive or complimentary interactive function, which is obviously difficult or not possible to achieve by human tutors [19].

Chatbot systems utilize linked and structured information in the semantic web for providing support to develop interactions in various works [20]. An advanced metaphysical model called OnBot [21] is taken as the basis for designing and developing efficient chatbots. In order to provide efficient chats, OnBot converts knowledge and metaphysics into relational datasets using the proper mapping approaches. With the help of the Egyptian rabic dialect, the Bota [22] chatbot investigates the difficulties in developing a user-friendly conversational tool. The use of the methods like Sequence-to-Sequence (Seq2Seq) and Information Retrieval (IR) are adopted and combined by the open-domain AliMeChat [23] chatbot engine. A multi-agent chatbot engine called Octopus [24] uses eight sub-multi-agent systems a system for Graphical User Interface (GUI), a system for core, a system for communication, a system for Natural Language Processing (NLP), a system for action, a system for accessing data, a system for learning, and a system for searching. In order to engage users in negotiation, a semi-automated social chatbot called SOGO [25] is an artificial negotiation dialogue-creating tool that combines conversational tactics with task utterance. By utilizing diverse user conversations as the base, these chatbots create and connect advanced conversation engines to communicate with clients in a natural way wherever they are used. Additionally, they offer a lot of real-world scenarios according to third-party data customization. But, the majority of intelligent chatbot engines have drawbacks regarding user privacy, the ability to comprehend natural questions, their designs, and moral dilemmas. While many concerns are discussed and resolved, some still remain unsolved. So, deep learning-based student intent-based chatbot is implemented to provide and help students with suitable replies to their queries.

The following list includes this paper's main features.

• To design an automatic and advanced student intent-based chatbot system for helping the students in clarifying their queries regarding their learning process and ease their process of interaction.
• To design an optimized AA-DTCN-SC by tuning or optimizing the elements in the AA-DTCN-SC model by the ADT-BMO algorithm so that an accurate and precise intent-based chatbot is developed.

To evaluate the performance of the proposed model by comparing it with other existing methods, algorithms, and chatbots.

## Related works

In 2020, Nuruzzaman and Khadeer [1] have recommended IntelliBot, a domain-based chatbot. This IntelliBot generated replies based on the dialogues used in the chatbot with the help of various techniques. In order to gain domain-specific expertise, IntelliBot was trained on two datasets that were created by custom-built insurance and a dataset created using the conversation from the Cornell movie. Following that, chatbots such as DeepQA, ChatterBot, and RootyAI from the existing works were used to validate and compare IntelliBot's performance. The outcomes showed the superior performance of IntelliBot in the field of insurance when it comes to developing an effective conversation with the user and giving a thorough response.

In 2021, Jaiwai et al. [2] have created a strategy for designing a chatbot-based question-answering system. The chatbots were incredibly user-friendly and intuitive as they used NL for human-machine communication. The goal of this study was to create a Thai question-and-answer system with the utilization of chatbot technology that was then coupled with a mobile app called LINE. Integration of Deep Neural Networks (DNN) and NLP were utilized for conducting experiments with the inclusion of a term from the educational system to the dictionary, and then word segmentation for three different text classification methods was compared.

In 2020, Rajkumar and Ganapathy [3] have discovered a connection between the learning preferences of individuals who were extroverts and introverts. At first, with the aim of categorizing the individuals as extroverts and introverts, a chatbot was used with the utilization of modified VARK. Following the categorization of the individuals as extroverts and introverts by the chatbot, a dataset containing the beta brain waves of the learners, introverts, and extroverts was recorded and stored at one-second intervals by providing two-minute audio-video data. Popular machine learning approaches like the N48, Canopy, and Naive Bayes were utilized to validate and compare the efficiency of the generated dataset. It was discovered that the suggested strategy increased the learner’s classification accuracy. A recommendation system called the Bio-Inspired Learning Style Brain Computing Interface (BIL-BCI) model was also introduced to improve the categorization accuracy between the e-learners.

In 2023, Yao-Ping et al. [5] have developed a model for validating the cognitive usage and the attitude of university students in English M-learning by combining the Stimulus Organism Response framework with the original technological acceptance framework, which includes the notions of curiosity, self-efficacy, and perceived suitability. In order to analyze the framework’s design and validate the assumptions, this work used Smart-PLS 4.0. For aiding in usage, a positive and significant relationship between attitudes towards using, perceived ease of use, and perceived usefulness were important, whereas the intention of using was positively impacted by self-efficacy, intention to use, and curiosity. The experimental results of this work have also offered a wealth of theoretical explanations and practical implications for learning languages.

In 2023, Consuelo et al. [7] have created a sample of 42 undergraduate and 15 master's degree students in the field of health sciences from 57 students from various universities. A hybrid research approach was used. In the quantitative study, the effects of the level of education and the amount of existing knowledge were examined in relation to learning results, satisfaction, and chatbot usage frequency with the chatbot's utility. Additionally, they looked into whether or not students' metacognitive techniques affected how frequently chatbots were used by them. The qualitative study examined the ideas made by the students for enhancing the chatbot and the kinds of questions it needed to be asked. The findings showed that levels of prior knowledge only had an effect on learning outcomes, with Master's degree students using chatbots more frequently and achieving better levels of learning outcomes. The perceived pleasure of the students with the chatbot's use also showed significant differences, with master's students ranking higher, but not in relation to their existing knowledge levels. Regarding the frequency at which the chatbot was used and the student's metacognitive skills level, no clear findings were discovered. Based on the students' recommendations for enhancement, additional investigations are required to direct this research work.

In 2022, Mageira et al. [8] have designed AsasaraBot, which was an experimental user model of an educational AI chatbot with the goal of teaching cultural content to high school pupils in either French or English language. The Minoan Civilization was mentioned in the provided text, content with a focus on the distinctive statue of the Snake Goddess of the Minoan. Greek public and private language schools have examined the relevant chatbot-based instructional program. The results of these studies demonstrated that interactive ICT-based learning using AI chatbot technology was appropriate for simultaneously learning foreign cultures and languages. Using free and open-source software, the AsasaraBot AI chatbot was developed and executed into use as part of a postgraduate project.

## Method details

### Implemented student intent-based educational chatbot system

**Fig. 1:**
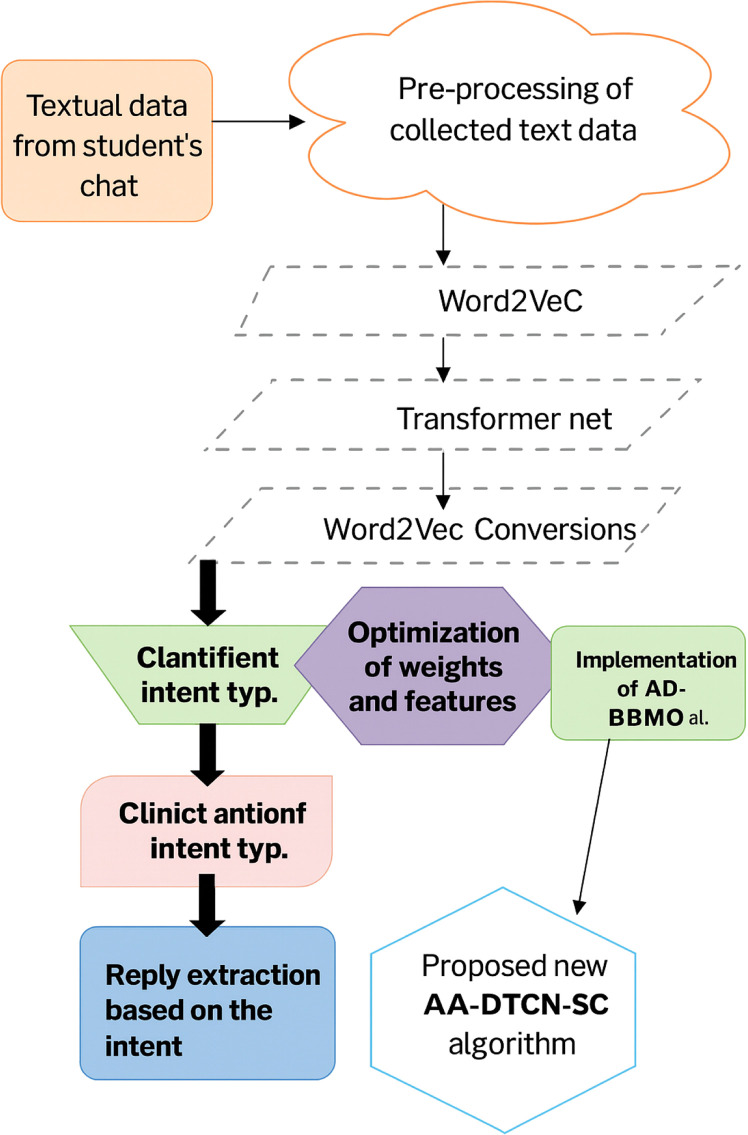
Illustration of the generated student intent-based educational chatbot.

Even though many chatbots have been recently developed, none of them is able to provide suitable replies for all the queries asked by the student. No chatbot that currently exists in the market can be able to provide friendly interaction with students. Also, none of the chatbots can be used for multilingual purposes. This leads to irrelevant replies when queries are asked in the local language or when there occur a spelling mistake in the query asked. So, an efficient student intent-based chatbot needs to be developed. In our work, a deep learning-based student intent-based chatbot is designed for solving the above-mentioned issues. The illustration of the generated Student Intent-based Educational Chatbot system is shown in Fig. 1.

The suggested architecture is a strong and clever system of student chat bots which should understand, label and answer academic questions with the combination of deep learning models and a hybrid optimization algorithm. This pipeline starts with the process of ingesting student chatbot data consisting of questions on syllabus, exams, elementary information, and other academic fields. Text Preprocessing is the initial part of this system, and it is vital in the cleansing and normalization of the unprocessed textual data. The stage involves three key procedures namely removal of special characters, stemming (cutting words to their root), and removal of punctuations. Such operations have to be done to diminish the noise and normalize the format according to which it is to be handled by the next deep learning algorithms. The clean textual data in the form of vector representations is then created after preprocessing them. It will be done through three models of a natural language namely, Text Convolution Neural Network (Text CNN), Bidirectional Encoder Representations from Transformers (BERT), and Transformer Net. Every model possesses its own unique advantages [39], Text CNN represents local semantic patterns, BERT represents the directional relationship between words, and Transformer Net imposes the best performance in terms of self-attention when dealing with sequences. Such models produce dense representations that obtain both syntactic and semantic meanings so that the system can interpret the subtle nature behind human requests better. These models will then give the outputs which are subsequently standardized by a Word2Vec Conversion that allows compatibility and the uniformity of the used vectorized format. These feature vectors are not utilized discreetly rather features are fused with weighted schemes to fabricate unified and richer set of features.

It is attained by utilizing a new algorithm called Averaging-based Driving Training-Barnacles Mating Optimizer (ADT-BMO) since it has two functionalities; it not only achieves the selection of the most optimal features in the output of each model but also it assigns these features with weights that can maximize the correlation and eliminate redundancy [40]. The end result of this stage is a Weighted Fused Feature Vector that contains the most discriminative and relevant attributes regarding the classification task. Such consolidated properties are forwarded to the centerpiece of the architecture: the Adaptive and Attentive Deep Temporal Convolution Network with Symmetric Convolution (AA-DTCN-SC). Besides, the model has its own attention mechanism and prioritization of certain segments of the input, making the model more interpretable and accurate. The ADT-BMO algorithm can help once again at this point by tuning the important hyper-parameters like the amount of hidden neurons, choice of the activation functions, and amount of training epochs. This allows us to have the AA-DTCN-SC model not only extremely powerful but also efficient and well-customized to execute on the specific dataset. Then the trained model enters the process of Intent Classification, in which each question by a student is assigned to one of several pre-determined categories- such as welcome messages, internal marks, subject-related questions, etc [41]. The result of the classification is then directed into the Reply Extraction module that then searches or creates a relevant and informative response depending on the identified intent. This guarantees the students the correct response at the time they ask questions and it also keeps them away from relying on the administrative staff and makes the institution more responsive. The architecture solves major problems within chatbot systems as to relevancy of answers, slowness in response and poor intent recognition through a combination of multi-model vectorization, intelligent optimization and powerful deep learning [42]. The application of numerous models produces semantic richness; the ADT-BMO algorithm produces relevance and brevity and the AA-DTCN-SC model produces contextual understanding. In combination we would get a scalable, understandable, and efficient chatbot framework that could be used in an academic setting [35].

### Proposed ADT-BMO

The process of optimization of the hidden neurons of the AA-DTCN-SC, epochs of the AA-DTCN-SC, activation function of the AA-DTCN-SC, along with the optimization of the vectors from the BERT, TransfromerNet, and Text CNN, and the optimization of the weights for performing the weighted feature fusion is done with the aid of the suggested ADT-BMO algorithm [28]. The enhanced efficiency and the improved performance make us choose the DTBO for our work. But, the real-world application of the DTBO algorithm is low [29]. The BMO algorithm is utilized because of its unimodal global optimum, multimodal exploration, and its capability to prevent local optimum in composite operations. Yet, the inaccurate search function remains the drawback of this algorithm. So, the DTBO algorithm is fused with the BMO algorithm to improve its benefits [30]. In the recommended ADT-BMO algorithm, the location update takes place by implementing the fitness-based concept as given in Eq. (1).

(1) $$pos=\frac{mean\left(pos1,pos2\right)+\min\left(pos1,pos2\right)}{100}$$

In Eq. (1), the termpos1denotes the position update using the DTBO algorithm,pos2denotes the position update using the BMO algorithm,meandenotes the mean value, andmindenotes the minimum value.

DTBO [26]: The way a novice learns to drive has influenced how the DTBO algorithm was created. The novice initially picks an instructor from among the many available tutors at the driving school. The coach then shows the learner how to operate the vehicle and gives instructions [35]. The learners' ongoing, repeated practice then aids in the development of their skills. This serves as the backbone in the development of the DTBO algorithm [43]. Like many other population-based algorithms, the DTBO algorithm is one as well. In the DTBO algorithm, the population includes both the instructor and the beginners. Eq. (2) is used in the DTBO algorithm to compute the population vector [36].

(2) $$M={\left[\begin{array}{c} M_{1}\\ \vdots \\ M_{l}\\ \vdots \\ M_{s} \end{array}\right]}_{s\times v}={\left[\begin{array}{ccccc} m_{11} & \cdots & m_{1r} & \cdots & m_{1v}\\ \vdots & \ddots & \vdots & \ddots & \vdots \\ m_{o1} & \cdots & m_{or} & \cdots & m_{ov}\\ \vdots & \ddots & \vdots & \ddots & \vdots \\ m_{s1} & \cdots & m_{sr} & \cdots & m_{sv} \end{array}\right]}_{s\times v}$$

In Eq. (2), the termMis the representation of the total individual count,sis the representation of the total candidate in the entire algorithm,vis the representation of the entire variable count, andois the representation of the candidate solution. The random initialization of the individual’s location is given by Eq. (3).

(3) $$m_{o,r}=w_{r}+x\left(y_{r}-w_{r}\right);\;o=1,2,..,s;\;\;r=1,2,..,v$$

The termyin Eq. (3) is the representation of the problem’s upper limit andwis the representation of the problem’s lower limit [37]. For every solution, the evaluation of the fitness constant is done with the help of Eq. (4).

(4) $$z={\left[\begin{array}{c} z_{1}\\ \vdots \\ z_{l}\\ \vdots \\ z_{s} \end{array}\right]}_{s\times 1}={\left[\begin{array}{c} z\left(M_{1}\right)\\ \vdots \\ z\left(M_{o}\right)\\ \vdots \\ z\left(M_{s}\right) \end{array}\right]}_{s\times 1}$$

In Eq. (4), the termzis the representation of the fitness function vector for the entire solution. The ideal solution in the DTBO$M_{D}$is the one with the best fitness. Every iteration ends with the amendment of the ideal solution [44]. The key vital procedures that the DTBO algorithm undergoes for updating the ideal solution are given below.

1) Training for the novice by the instructor
2) Learning the key techniques
3) Repeated practicing

Training for the novice by the instructor: The student selects a specific driving school instructor to serve as their mentor during the primary stage of learning. The novice is given access to all the best driving abilities that the instructor possesses by the chosen instructor. It is taken as the first step of the DTBO algorithm [37]. A person's best qualities are chosen to serve as instructors, while the balance is used as students in the DTBO algorithm. The choice of the instructor and learning from the instructor causes the learner to explore more locations of the lookup area. This stage is called the exploring phase. The exploration capabilities of the DTBO algorithm are improved, and this facilitates global searching for the optimal location in the lookup region. As demonstrated in Eq. (5), the DTBO algorithm chooses the instructor at the start of each cyclesfrom among those with the optimum fitness levels.

(5) $$A=\;{\left[\begin{array}{c} A_{1}\\ \vdots \\ A_{o}\\ \vdots \\ A_{s_{A}} \end{array}\right]}_{s_{A}\times v}\;=\;{\left[\begin{array}{ccccc} A_{11} & \cdots & A_{1r} & \cdots & A_{1v}\\ \vdots & \ddots & \vdots & \ddots & \vdots \\ A_{o1} & \cdots & A_{or} & \cdots & A_{ov}\\ \vdots & \ddots & \vdots & \ddots & \vdots \\ A_{s_{A}1} & \cdots & A_{s_{A}r} & \cdots & A_{s_{A}v} \end{array}\right]}_{s_{A}\times v}$$

In Eq. (5), the term$s_{A}$is the representation of the instructor population andAis the representation of the matrix of instructors. Eq. (6) provides the population of the instructor$s_{A}$.

(6) $$s_{A}=\left[0.1\cdot s\cdot \left(1-\frac{q}{Q}\right)\right]$$

The termQin Eq. (6) is the representation of the highest iteration that is being executed in the DTBO algorithm andqis the representation of the ongoing iteration. Eq. (7) provides the location for every candidate.

(7) $$m_{o,r}^{B1}=\left\{ \begin{array}{cc} m_{o,r}+x\cdot \left(m_{o,r}-A_{Eo,r}\right) & z_{A_{Eo}}>z_{o}\\ m_{o,r}+x\cdot \left(A_{Eo,r}-F\cdot m_{o,r}\right) & z_{A_{Eo}}<z_{o} \end{array}\right.$$

In Eq. (7), the termFis the representation of a randomized variable between{1,2}and$A_{Eo,r}$is the representation of the randomly picked instructor for giving the essential coaching to the noviceofrom$\left\{1,2,...,s_{A}\right\}$. Eq. (8) is used to amend the location provided if the old one has the least fitness than the new one.

(8) $$M_{o}=\left\{ \begin{array}{cc} M_{o} & z_{o}^{B1}>z_{o}\\ M_{o}^{B1} & z_{o}^{B1}<z_{o} \end{array}\right.$$

The term$M_{o}^{B1}$ in Eq. (8) is the representation of the new location for the$o^{th}$candidate, Bis the representation of the variable of pattern, and$z_{o}^{B1}$ is the representation of the fitness. Eq. (9) provides the variable of the pattern.

(9) $$B=\frac{10}{1000}+\frac{18}{20}\left(1-\frac{q}{Q}\right)$$

Learning the key techniques: The novice attempts to acquire the skills from their instructor during the second phase of exploration. At this phase, the novice replicates their instructor's driving style and their full skill [45]. This improves the DTBO's capacity for exploration and encourages DTBO members to discover new areas inside the search region. As demonstrated in Eq. (10), an improved location is created by linearly integrating each individual novice and their instructors [38].

(10) $$m_{{}_{o,r}}^{B2}=B\cdot m_{o,r}+\left(1-B\right)\cdot A_{Eo,r}$$

Eq. (10) is regarded aspos1in Eq. (1). Provided that the old location has a fitness value much lesser than the new one, then based on Eq. (11), the new location upgrade takes place.

(11) $$M_{o}=\left\{ \begin{array}{cc} M_{o} & z_{o}^{B2}>z_{o}\\ M_{o}^{B2} & z_{o}^{B2}<z_{o} \end{array}\right.$$

The term$z_{o}^{B2}$is the representation of the fitness and$M_{o}^{B2}$ is the representation of the new location for the candidate$o^{th}$at this exploration phase.

Repeated practicing: The primary task carried out in this stage of the DTBO algorithm is the practice that each novice undertakes with the intention of enhancing and improving their driving skills. Each novice advances at this level with the intention of acquiring the best skills possible. As this is the local exploitation phase, each novice attempts to determine new destinations nearby based on their current location by conducting local searches. To carry out the local exploitation, an arbitrary site is initially computed closer to each person. Eq. (12) provides the arbitrary location that was formed.

(12) $$m_{o,r}^{B3}=m_{o,r}+\left(1-2x\right)\cdot C\cdot \left(1-\frac{q}{Q}\right)\cdot m_{o,r}$$

Provided that the old location has a fitness value much lesser than the new one, then based on Eq. (13), the new location upgrade takes place.

(13) $$M_{o}=\left\{ \begin{array}{cc} M_{o} & z_{o}^{B3}>z_{o}\\ M_{o}^{B3} & z_{o}^{B3}<z_{o} \end{array}\right.$$

The term$z_{o}^{B3}$in Eq. (13) is the representation of the fitness,$M_{o}^{B3}$ is the representation of the new location for the candidate$o^{th}$at the exploitation phase, andCis a fixed term with a value of$\frac{1}{2}$. The locations are upgraded at every iteration until it reaches the highest possible iteration. The best location is obtained at the end of the maximum iteration.

BMO [27]: There are three main stages that are performed by the BMO algorithm. They are initializing the parameters, selecting the choice, and mating. The execution of the above-mentioned stages is provided below.

Initializing parameters: The population of the Barnacle considered by the BMO algorithm is initialized, which is given by Eq. (14).

(14) $$a=\left[\begin{array}{ccc} c_{1}^{1} & \cdots & c_{1}^{d}\\ \vdots & \ddots & \vdots \\ c_{f}^{1} & \cdots & c_{f}^{d} \end{array}\right]$$

The termfin Eq. (14) denotes the barnacle count andddenotes the count of the variables that control the process. The process of estimating the value ofais the initial process. The arrangement stage is done to find the optimum solution. The boundaries of the lookup area are given by (15), (16).

(15) $$i_{h}=\left[i_{h}^{1},...,i_{h}^{j}\right]$$

In Eq. (15), the term$i_{h}$denotes the upper boundary at$j^{th}$ element.

(16) $$g_{h}=\left[g_{h}^{1},...,g_{h}^{j}\right]$$

In Eq. (16), the term$g_{h}$denotes the lower boundary at$j^{th}$element.

Selection of choice: The reproductive behavior of the Barnacle in choosing its mate is imitated by the BMO algorithm. Based on the size of the male’s penisl, the female Barnacle chooses its mate. The below-provided steps explain the assumptions that are imitated by the behavior of the female Barnacle in choosing its mate.

The process in which the female Barnacle chooses its mate is arbitrary, but it is limited to the size of the male’s penisl.

Even if the female Barnacle is fertilized by many male Barnacles in real-time, we assumed that only one male is paired with one female at a time.

The process of self-mating is avoided in this work. Thus, no young ones are produced by this process.

The process of casting sperm occurs if the size of the penis at any iteration becomes larger than the chosen sizel.

The first two points in the assumption infer the exploitation process, and the last point in the assumption infers the exploration process. The ideal candidate is present in the upper section of the solution matrix. The maximum size of the Barnacle’s penis is 7. So, a barnacle can mate with only a barnacle within this limit. If a barnacle tries to mate outer than this limit, then the process of casting the sperm takes place. Since the actual distance of separation between the barnacles is unknown, only the virtual distance is considered in the BMO algorithm. The process of pair selection is explained by (17), (18).

(17) $$b_{e}=p\left(f\right)$$

(18) $$b_{n}=p\left(f\right)$$

In (17), (18), the parents that are chosen for mating are denoted by$b_{n}$and$b_{e}$. The process of choosing the mating pairs is done randomly, as given by the first assumption.

Mating: The inheritance of the parent barnacles on creating young ones is obtained on the basis of the Hardy-Weinberg principle, as there is no particular equation to imitate the mating of the barnacles. Eq. (19) shows the computational simplicity of the BMO algorithm in generating new young ones.

(19) $$c_{j}^{newd}=tc_{b_{e}}^{d}+uc_{b_{n}}^{d}$$

In Eq. (19), the term$c_{b_{e}}^{d}$denotes the father barnacle,tdenotes an arbitrary variable in the range[0,1], and$c_{b_{n}}^{d}$denotes the mother barnacle. The terms *t*and *u* denotes the proposition in which the features of the mother and father barnacle are inherited by the young one. The value of *u* is obtained from *t* as u=1−t. The possibility in the young one inherits the characteristics of the father and mother barnacle is in the range[0,1]. The size of the penis plays a vital role in fixing the exploration and exploitation stage. When the chosen Barnacle for reproduction is within the limit of the father’s penis size, then Eq. (19) is carried out to perform exploitation. If the limit deviates, then exploration takes place by implementing the process of casting the sperm. Eq. (20) provides the process by which the sperm is cast.

(20) $$c_{j}^{newf}=k\left(\right)\times c_{b_{n}}^{f}$$

In Eq. (20), the termk()denotes an arbitrary function in the range[0,1]. Eq. (20) denotes the generation of the young ones by the barnacles. In the exploration stage, the young ones are produced by the mother barnacle as it is fertilized by the sperm of a random barnacle that is present in the water. The optimal solution is upgraded at the completion of every cycle, and it is present in the upper region of the solution matrix *a*. To maintain a balance of population matrix, the young ones are fused with their parents. The top candidate with the best fitness is arranged and chosen for the best solution, while the worst fitness candidates are eradicated. Based on the value ofl, Eq. (19) or Eq. (20) is considered aspos2in Eq. (1). The Pseudocode of the implemented ADT-BMO algorithm is given in Algorithm 1.

**Table:** Algorithm 1: Implemented ADT-BMO algorithm.

Load the population and parameters in BMO and DTBO algorithms				
Compute the fitness value for the entire population				
For(ab=1→Y)				
	For$\left(bc=1\to Z_{\max}\right)$			
		While(terminating condition is met)		
			The location is updated using the DTBO algorithm	
			Perform the primary exploration phase	
			Perform secondary exploration phase	
			Perform exploitation phase	
			Upgrade the position1pos1	
			The location is updated using the BMO algorithm	
			If(l≥7)	
				Perform exploration phase Eq. (19)
			Else	
				Perform exploitation phase Eq. (20)
			Upgrade position 2pos2	
			End	
			Position update is done by utilizing the adaptive concept given in Eq. (1)	
			The fitness of the optimal position is calculated	
			The best position is upgraded	
		End		
	End			
End				

**Fig. 2:**
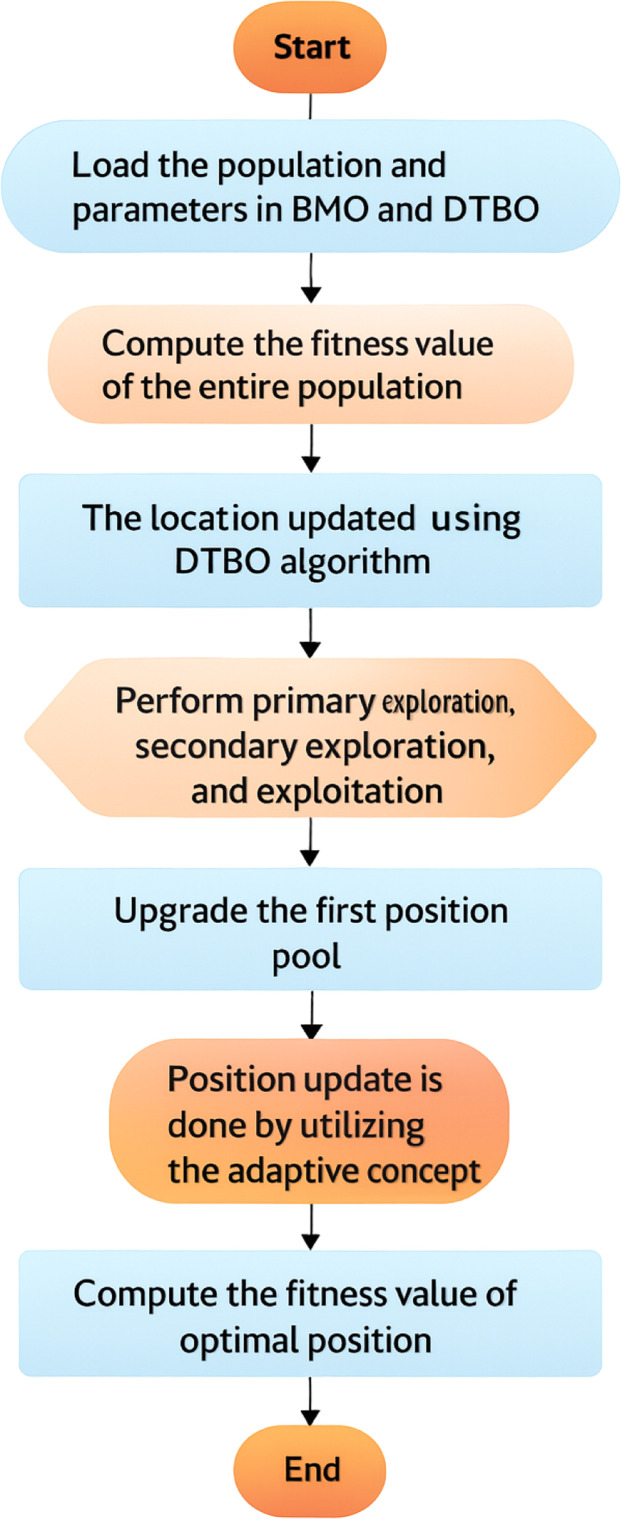
Flowchart of the implemented ADT-BMO algorithm.

The flowchart of the implemented ADT-BMO algorithm is given in Fig. 2.

## Method validation

### Proposed ADT-BMO-based weighted feature fusion

The acquired vector representation of the pre-processed texts of BERT, TransformerNet, and Text CNN can now be regarded as the basis of the weighted feature selection and fusion. Initially there is the selection of the best attributes of the vector form of words of BERT, TransformerNet and Text CNN using developed ADT-BMO algorithm. Represented by $OC_{fs}^{BERT}$ is the optimum characteristic attained with BERT. The chosen best feature of the TransformerNet is presented by $OC_{ft}^{TRAN}$. And the corresponding optimal feature selected of the Text CNN can be schematized as $OC_{fr}^{TCNN}$. The same ADT-BMO algorithm is also optimized to two weights $X_{1}$and $X_{2}$. The best attributes are selected now. This is then combined together with the optimized weights to form the weighted fused features. The calculation of the weighted fused features is as below in Eq. (21), (22).

(21) $$FF_{we}^{fusion}=\left(X1*OC_{fs}^{BERT}\right)+\left(\left(1-X1\right)*\left(OC_{ft}^{TRAN}\right)\right)$$

(22) $$WF_{ud}^{fiuse}=\left(X2*FF_{we}^{fusion}\right)+\left(\left(1-X2\right)*OC_{fr}^{TCNN}\right)$$

In Eq. (26) the term$WF_{ud}^{fiuse}$ represents the weighted fused feature. The aim of doing the weighted feature fusion is to optimize the correlation coefficient between the characteristics, which is expressed as Eq. (23)

(23) $$AB1=\underset{\left\{OC_{fs}^{BERT},OC_{ft}^{TRAN},OC_{fr}^{TCNN},X1,X2\right\}}{\arg\;\;\min}\;\left(\frac{1}{LK}\right)$$

In the Eq. (24), the term AB1represents the objective function, task-selected vectors in BERT are represented by $OC_{fs}^{BERT}$, optimally chosen vectors of the limit task, optimally chosen vectors of in the limit task, optimally chosen vectors of task which is in the limit [1,50], $OC_{ft}^{TRAN}$optimally chosen vectors of which is in the limit [1,50], $OC_{fr}^{TCNN}$and tuned weights which is in the limit represented by [0.01,0.99]and LKrepresents the correlation coefficient. The correlation coefficient is specified by the Eq. (24)

(24) $$LK=\sum \left(WF_{ud}^{fuse}-\;mean\left(WF_{ud}^{fuse}\right)*\;\frac{WF_{ud}^{act}-\;mean\left(WF_{ud}^{act}\right)}{dc\;*\;\sigma \left(WF_{ud}^{fuse}\right)\;*\;\sigma \left(WF_{ud}^{act}\right)}\right)$$

**Fig. 3:**
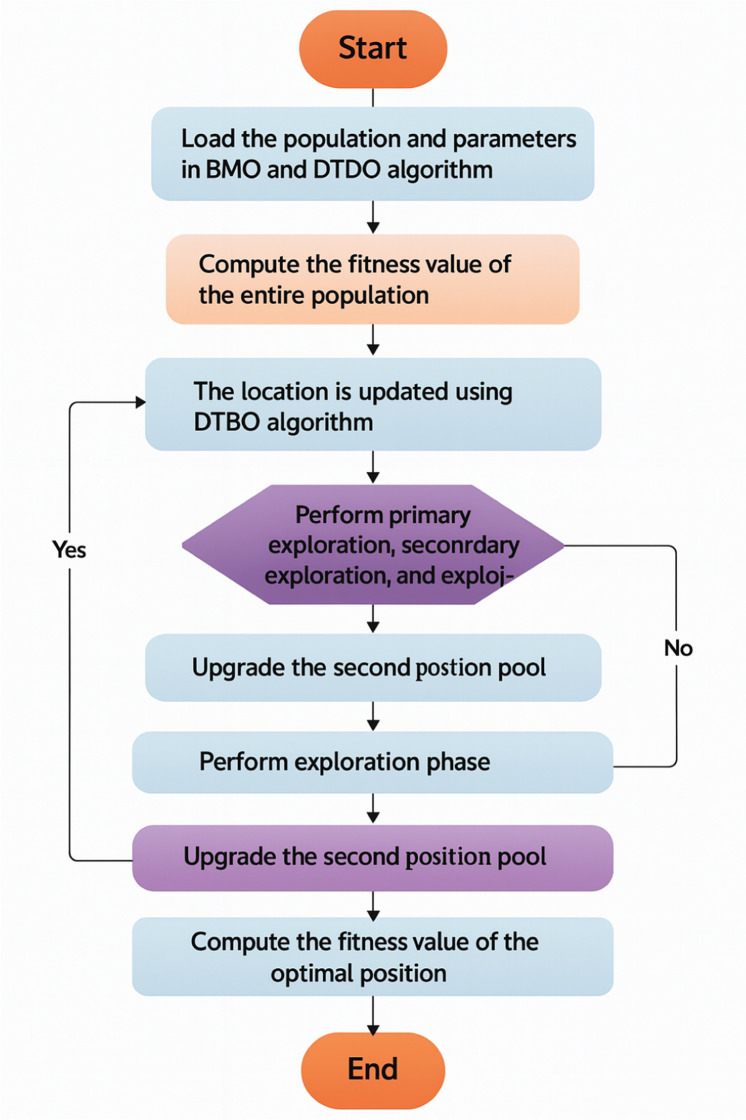
Proposed approach with some basic parameters.

In Eq. (24) the term $WF_{ud}^{act}$is the actual weighted fused features, dc denotes the sample size, and is the Standard deviation representation. Fig. 3 presents pictorial explanation of process of fusion of weighted features

### Student chatbot dataset

The dataset obtained from various standard educational chatbots is considered for developing the recommended student intent-based chatbot system. The data are collected from two chatbots, namely, the student chatbot and the student Tanglish chatbot. This dataset consists of the questions asked and the categorization of the question into various classes. A total of seven classes, namely, welcome message, basic information, subject, syllabus, internal 1, internal 2, and internal 3, are there in both datasets. The sample questions for each class in the provided dataset are given in Table 1. The obtained chatbot data are collectively represented as$CB_{da}^{data}$.

**Table 1:** Sample chatbot data from the collected dataset.

Dataset/ Classes	Student Chatbot	Student Tanglish Chatbot
Welcome message	Hello! Welcome to our Chatbot Hi there, how can I help?	வணக்கம் ! எங்கள் ChatBot க்கு வரவேற்கிறோம் வணக்கம், நான் எப்படி உதவுவது?
Basic Information	We need your Date of Birth Enter Your mother tongue	ungal name enna entha degree neenga
Subject	What are the core papers in the ECE branch Any machine learning course conducted by the CSE department	ennena core papers irukku ECE branch How do I sign up for the basketball club
Syllabus	When will I submit the project report what are the chapters in ge8151	When will they issue the hall ticket How many internal exams are there
Internal 1	examination duration final exam date	when comes results give model question paper
Internal 2	The procedure of assessment of theory answer book? Was the answer sheet shown to the student?	Internal Assessment is conducted as per COE guidelines. Was the answer sheet shown to the student?
Internal 3	what are basic pass marks how can I prepare for my exam	Is there any session regarding exam discussion Give the timetable for internal exam 3

### Activation function-based performance evaluation on designed student intent-based chatbot with traditional algorithms

**Fig. 4:**
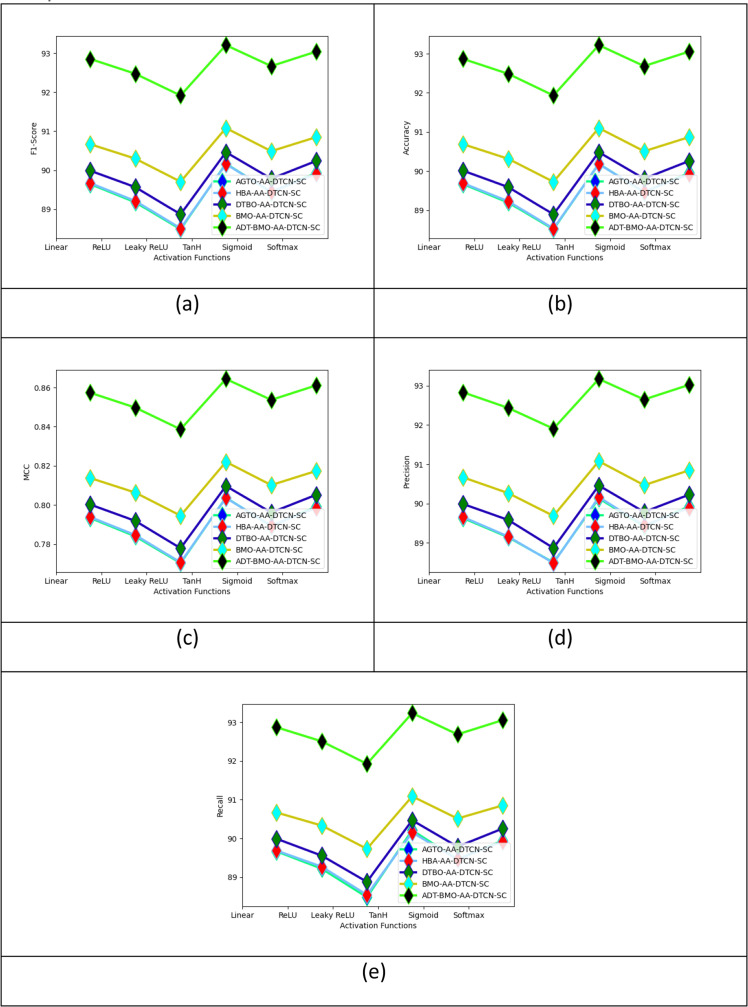
Activation function-based performance evaluation of the designed student intent-based chatbot with traditional algorithms in terms of “ (a) F1-score, (b) accuracy, (c) MCC, (d) precision, and (e) recall”.

The activation function-based performance evaluation of the designed student intent-based chatbot with traditional algorithms is given in Fig. 4. The evaluation of the system with varying the activation function helps in maintaining linearity while conducting the experiments. For the ReLU activation function, the accuracy of the designed ADT-BMO-AA-DTCN-SC Student Intent-based Chatbot is 2.54%, and 3.22% improved than the BMO-AA-DTCN-SC, and DTBO-AA-DTCN-SC algorithms, and 3.69% higher than both HBA-AA-DTCN-SC and AGTO-AA-DTCN-SC algorithms, respectively.

### Chatbot-based examination of the executed student intent-based chatbot

**Fig. 5:**
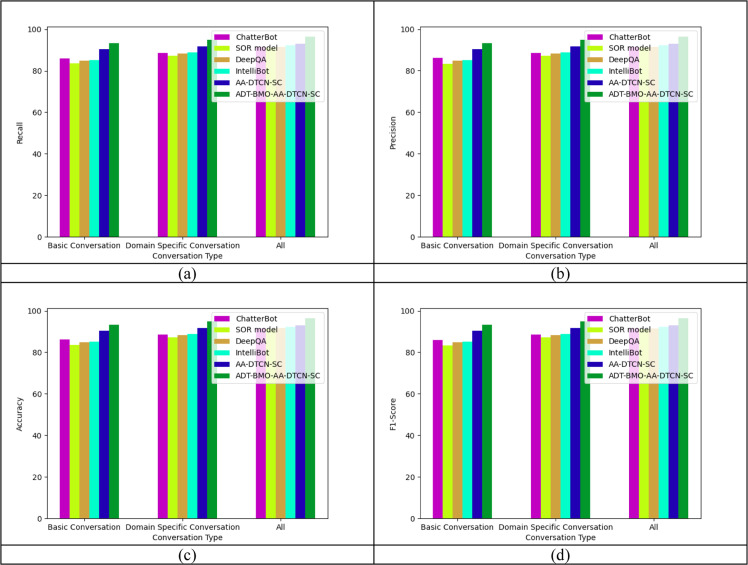
Performance assessment of the implanted student intent-based chatbot with conventional classifiers based on feature-based assessment in terms of (a) recall, (b) precision, (c) accuracy and (d) F1-score.

The chatbot-based examination of the executed student intent-based chatbot is provided in Fig. 5. The comparison of these chatbots is done by varying the type of conversation. This analysis on varying the type of conversation helps in identifying the efficiency of the chatbots in various applications. The executed ADT-BMO-AA-DTCN-SC Student Intent-based Chatbot is 0.22%, 1.35%, 1.69%, 4.88%, and 2.5% more accurate than the AA-DTCN-SC, IntelliBot, DeepQA, SOR model, and ChatterBot, respectively, for domain-specific conversation type.

### Classifier evaluation of the generated student intent-based chatbot

Table 2 shows the classifier performance of the produced student intent-based chatbot. The FPR of the produced ADT-BMO-AA-DTCN-SC Student Intent based Chatbot is 28.04, 30.37, 30.53 and 22.92 percent lower than AA-DTCN-SC, DTCN, Bi-LSTM and RNN classifications respectively.

**Table 2:** Classifier evaluation of the generated student intent-based chatbot.

Measures/ Classifiers	AA-DTCN-SC [32]	DTCN [31]	Bi-LSTM [33]	RNN [34]	ADT-BMO-AA-DTCN-SC
Specificity	82.887	81.282	77.210	82.879	92.681
FPR	17.113	18.718	22.790	17.121	7.319
Recall	82.895	81.276	77.217	82.923	92.687
FNR	17.105	18.724	22.783	17.077	7.313
Precision	82.824	81.212	77.131	82.822	92.650
FDR	17.176	18.788	22.869	17.178	7.350
NPV	82.958	81.346	77.294	82.980	92.718
F1-Score	82.860	81.244	77.174	82.873	92.669
Accuracy	82.891	81.279	77.213	82.901	92.684

**Fig. 6:**
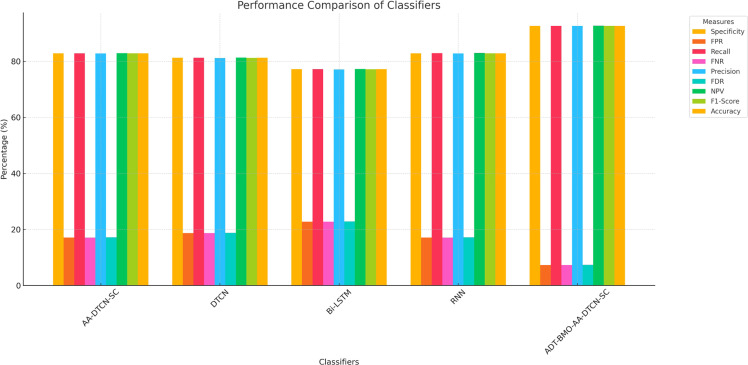
Compartive analysis of different classifiers.

The bar graph shows in Fig. 6 the comparative results of five classifiers-AA-DTCN-SC, DTCN, Bi-LSTM, RNN, and proposed ADT-BMO-AA-DTCN-SC, in terms of prominent evaluation parameters, i.e., accuracy, precision, recall, specificity, F1-score, etc., and error rates, like FPR, FNR, and FDR, etc. Regarding all of them, the proposed one, the ADT-BMO-AA-DTCN-SC model, performs extraordinarily better, getting more than 92 percent on most positive metrics and preserving the most minimal error rates. To exemplify the issue, its Accuracy (92.684%), Recall (92.687%), and F1-Score (92.669%) are the largest of them all. Bi-LSTM, instead, records the lowest scores in all the four measures, which have a limitation of below 78 percent. The experimental findings show the competence of feature fusion and optimization through ADT-BMO algorithm which increases model robustness and intent classification. AA-DTCN-SC and RNN go relatively well but cannot compete with the enhanced model of ADT-BMO. The proposed system has a low value of FPR (7.319%), FNR(7.313%), which means the system will be accurate to avoid false predictions. In sum, it can be concluded further that the combination of deep temporal convolutional learning and the hybrid optimization has delivered better results in an educational chatbot.

## Discussion

A Smart and Automated Student Intent-based Educational Chatbot was also designed and developed, and it helped the students in getting the academic information on time. This was initiated by gathering the data about student conversation by use of common chat-bots data sets. To analyze this raw text further, its pre-processing was carried out using such methods as cleaning, stemming, and all punctuations removal. Three strong models, that is, BERT, TransformerNet and Text CNN, were used to convert the cleaned data into a form of vector. These Vectors were optimized by performing a weighted feature selection and fusion process, optimally selected and weighted by the new ADT-BMO algorithm which intelligently selected and weighted features. The resultant output after fusing was subsequently inputted into the AA-DTCN-SC in order to classify the intent. ADT-BMO also performed the tuning of hyperparameters of the model using accuracy and precision. According to the category intentions, proper responses were developed to the student queries. Simulation findings showed that the proposed system had competitive advantages over the other known models (AA-DTCN-SC, DTCN, RNN, and Bi-LSTM) in the relative ratios of 4.44 percent, 3.3 percent, 10.59 percent and 11.9 percent respectively. This shows the effectiveness of the chatbot, which provides effective and timely academic assistance that is context-sensitive.

## Limitations

None.

## Ethics statements

None.

## Funding

The authors received no funding for this work.

## Credit author statement

**Atul Kathole:** Methodology, Software, Conceptualization, Writing – original draft. **Suvarna Patil:** Data curation, Methodology, Visualization, Writing – original draft. **Dr. Devyani Jadhav:** Project administration, Methodology, Writing – review & editing, Funding acquisition. **Hirkani Pathak:** Methodology, Visualization, Writing – review & editing. **Amita Sanjiv Mirge:** Methodology, Conceptualization, Writing – review & editing.

## Declaration of competing interest

The authors declare that they have no known competing financial interests or personal relationships that could have appeared to influence the work reported in this paper.

## Data Availability

Data will be made available on request.

## References

[1] Mohammad Nuruzzaman, and Omar Khadeer Hussain, “IntelliBot: a dialogue-based chatbot for the insurance industry,” Knowl. Based Syst., Vol. 196, no. 105810, 21 May 2020.

[2] Jaiwai M., Shiangjen K., Rawangyot S., Dangmanee S., Kunsuree T., Sa-nguanthong A. (2021). Joint International Conference on Digital Arts, Media and Technology with ECTI Northern Section Conference on Electrical, Electronics.

[3] Rajkumar R., Ganapathy V. (2020). Bio-inspiring learning style chatbot inventory using brain computing interface to increase the efficiency of E-learning. IEEE Access.

[4] Ait-Mlouk A., Jiang L. (2020). KBot: a knowledge graph based ChatBot for natural language understanding over linked data. IEEE Access.

[5] Michael Yao-Ping Peng, Yunying Xu, and Cheng Xu,"Enhancing students' english language learning via M-learning: integrating technology acceptance model and S-O-R model," vol. 9, no. 2, Jan 31, 2023. 10.1016/j.heliyon.2023.e13302 PMC9900360 36755609

[6] Amantha Kumar Jeya (2021). Educationalchatbots for project-based learning: investigating learning outcomes for a team-based design course. Int. J. Educ. Technol. High. Educ..

[7] María Consuelo Sáiz-Manzanares, Raúl Marticorena-Sánchez, Luis Jorge Martín-Antón, Irene González Díez, and Leandro Almeida,"Perceived satisfaction of university students with the use of chatbots as a tool for self-regulated learning,"Vol. 9, no. 1, January 2023. 10.1016/j.heliyon.2023.e12843 PMC9871218 36704275

[8] Mageira Kleopatra, Pittou Dimitra, Papasalouros Andreas, Kotis Konstantinos, Zangogianni Paraskevi, Daradoumis Athanasios (2022). Educational AI chatbots for content and language integrated learning. Appl. Sci..

[9] Cai W., Jin Y., Chen L. (June 2022). Task-oriented user evaluation on critiquing-based recommendation chatbots. IEEE Trans. Hum. Mach. Syst..

[10] Daniel G., Cabot J., Deruelle L., Derras M. (2020). Xatkit: a multimodal low-code chatbot development framework. IEEE Access.

[11] Medeiros L., Bosse T., Gerritsen C. (June 2022). Can a chatbot comfort humans? Studying the impact of a supportive chatbot on users’ self-perceived stress. IEEE Trans. Hum. Mach. Syst..

[12] Wu E.H.-K., Lin C.-H., Ou Y.-Y., Liu C.-Z., Wang W.-K., Chao C.-Y. (2020). Advantages and constraints of a hybrid model K-12 E-learning assistant chatbot. IEEE Access.

[13] Cheng Z., Jiang Z., Yin Y., Wang C., Gu Q. (2022). Learning to classify open intent via soft labeling and manifold mixup. IEEE/ACM Trans. Audio Speech Lang. Process..

[14] Chen T.-Y., Chiu Y.-C., Bi N., Tsai R.T.-H. (2021). Multi-modal chatbot in Intelligent manufacturing. IEEE Access.

[15] Srivastava B., Rossi F., Usmani S., Bernagozzi M. (Dec. 2020). Personalized chatbot trustworthiness ratings. IEEE Trans. Technol. Soc..

[16] García-Méndez S., De Arriba-Pérez F., González-Castaño F.J., Regueiro-Janeiro J.A., Gil-Castiñeira F. (2021). Entertainment chatbot for the digital inclusion of elderly people without abstraction capabilities. IEEE Access.

[17] Zhang W., Yang F., Liang Y. (2019). A Bayesian framework for joint target tracking, classification, and intent inference. IEEE Access.

[18] Mazzei A. (Oct. 2022). Anticipating user intentions in customer care dialogue systems. IEEE Trans. Hum. Mach. Syst..

[19] Honda H., Hagiwara M. (2019). Question answering systems with deep learning-based symbolic processing. IEEE Access.

[20] Carlander-Reuterfelt D., Carrera Á., Iglesias C.A., Araque Ó., Sánchez Rada J.F., Muñoz S. (2020). JAICOB: a data science chatbot. IEEE Access.

[21] Al-Zubaide H., Issa A.A. (2011). International Symposium on Innovations in Information and Communications Technology.

[22] Ali Dana Abu, Habash Nizar (Dec 2016). Proceedings of COLING 2016.

[23] Qiu Minghui, Li Feng-Lin, Wang Siyu, Gao Xing, Chen Yan, Zhao Weipeng, Chen Haiqing, Huang Jun, Chu Wei (July 2017). Proceedings of the 55th Annual Meeting of the Association for Computational Linguistics.

[24] Hettige Budditha, Karunananda Asoka (November 2015). Proceedings of 8th International Research Conference.

[25] Zhao Ran, Romero Oscar J., Rudnicky Alex (November 2018). Proceedings of the 18th International Conference on Intelligent Virtual Agents.

[26] Dehghani Mohammad, Trojovská Eva, Trojovský Pavel (2022). A new human-based metaheuristic algorithm for solving optimization problems on the base of simulation of driving training process. Sci. Rep..

[27] Sulaiman MohdHerwan, Mustaffa Zuriani, Saari MohdMawardi, Daniyal Hamdan (January 2020). Barnacles mating optimizer: a new bio-inspired algorithm for solving engineering optimization problems. Eng. Appl. Artif. Intell..

[28] Futamata Kosuke, Park Byeongseon, Yamamoto Ryuichi, Tachibana Kentaro (26 Apr 2021). Phrase break prediction with bidirectional encoder representations in japanese text-to-speech synthesis. Interspeech.

[29] Sun J., Han P., Cheng Z., Wu E., Wang W. (2020). Transformer based multi-grained attention network for aspect-based sentiment analysis. IEEE Access.

[30] Kollias D., Zafeiriou S. (1 July 2021). Exploiting multi-CNN features in CNN-RNN based dimensional emotion recognition on the OMG in-the-wild dataset. IEEE Trans. Affect. Comput..

[31] Zhao W., Gao Y., Ji T., Wan X., Ye F., Bai G. (2019). Deep temporal convolutional networks for short-term traffic flow forecasting. IEEE Access.

[32] Tan Xianghua, Liu Yan, Liu Dandan, Zhu Dan, Zeng Weili, Wang Huawei (2022). An attention-based deep convolution network for mining airport delay propagation causality. Appl. Sci..

[33] Zhu Z., Yu H., Shen C. (March 2022). Waveform level intelligent multi-task receiver with BiLSTM. IEEE Commun. Lett..

[34] Kim J., Lee Y., Kim E. (2020). Accelerating RNN transducer inference via adaptive expansion search. IEEE Signal Process. Lett..

[35] Hashim Fatma A., Houssein Essam H., Hussain Kashif, Mabrouk Mai S., Al-Atabany Walid (February 2022). Honey badger algorithm: new metaheuristic algorithm for solving optimization problems. Math. Comput. Simul..

[36] Moradi Ehsan, Yaghoubi Behrouz, Shabanlou Saeid (2023). A new technique for flood routing by nonlinear Muskingum model and artificial gorilla troops algorithm. Appl. Water Sci..

[37] Gondek D.C. (May-June 2012). A framework for merging and ranking of answers in DeepQA. IBM J. Res. Dev..

[38] Schumaker R.P., Chen H. (January 2010). Interaction analysis of the ALICE chatterbot: a two-study investigation of dialog and domain questioning. IEEE Trans. Syst. Man Cybern. - A.

[39] Weihua L., Guo Y., Wang B., Yang B. (2023). Learning spatiotemporal embedding with gated convolutional recurrent networks for translation initiation site prediction. Pattern Recognit..

[40] Wu E.H.K., Lin C.H., Ou Y.Y., Liu C.Z., Wang W.K., Chao C.Y. (2020). Advantages and constraints of a hybrid model K-12 E-learning assistant chatbot. IEEE Access.

[41] Yanbu G., Zhou D., Li P., Li C., Cao J. (2022). Context-aware poly (a) signal prediction model via deep spatial–temporal neural networks. IEEE Trans. Neural Netw. Learn. Syst..

[42] Zhang W., Yang F., Liang Y. (2019). A Bayesian framework for joint target tracking, classification, and intent inference. IEEE Access.

[43] Zhao R., Romero O.J., Rudnicky A. (2018). Proceedings of the 18th International Conference on Intelligent Virtual Agents.

[44] Zhao W., Gao Y., Ji T., Wan X., Ye F., Bai G. (2019). Deep temporal convolutional networks for short-term traffic flow forecasting. IEEE Access.

[45] Zhu Z., Yu H., Shen C. (2022). Waveform level intelligent multi-task receiver with BiLSTM. IEEE Commun. Lett..

